# Death from Stings of Bees

**Published:** 1872-10

**Authors:** J. O. Sanders


RAPID DEATH FROM STINGS OF BEES.—J. O. Sanders, M. D., Reports the following: April 18th I was called to see a patient stung by bees. Mr. S., an intelligent man, gave the following account: Louis ——, a negro, aged about 45, climbed a tree where bees had swarmed on a limb, for the purpose of hiving them, carrying with him a saw. As soon as the limb commenced falling, the bees arose *en masse* and covered his head and face. He descended immediately, and, as soon as he reached ground, commenced running as fast as he could; ran around three sides of a yard, some two hundred steps, passed through an open gate, and fell to the ground. Mr. S. ran to him with a bottle of spts. camphor, and succeeded in forcing him to take one swallow; the patient protesting at the time against assistance, declaring that he would certainly die. After two or three irregular and partial respirations he expired. Mr. S. thinks it could not have been more than five minutes from the time he was attacked by the bees before he breathed his last. When I arrived, about an hour and a quarter after the accident, I could, on careful examination, find no signs of life. He was a vigorous, muscular man, and in perfect health, so far as I can learn. Was death due in this case to direct nervous shock, or to absorption of virus, or both?—*Medical News.*

